# Immunogenicity and safety of different combinations involving a third booster dose of SARS-CoV-2 inactivated vaccine, inactivated quadrivalent influenza vaccine, and 23-valent pneumococcal polysaccharide vaccine in adults aged ≥60 years: a phase 4, randomized, open-label study

**DOI:** 10.3389/fimmu.2024.1437267

**Published:** 2024-08-20

**Authors:** Shuang Bai, Shanshan Zhou, Junnan Zhang, Weixin Chen, Min Lv, Jian Wang, Ao Zhang, Jiang Wu, Wei Zhao

**Affiliations:** Beijing Center for Disease Prevention and Control, Beijing Research Center for Respiratory Infectious Diseases, Beijing, China

**Keywords:** inactivated SARS-CoV-2 vaccine, third booster dose, concomitant administration, influenza vaccine, 23-valent pneumococcal polysaccharide vaccine

## Abstract

**Background:**

Concomitant administration of COVID-19, influenza, and pneumococcal vaccines could reduce the burden on healthcare systems. However, the immunogenicity and safety of various combinations of a third booster dose of SARS-CoV-2 inactivated vaccine (CoronaVac), inactivated quadrivalent influenza vaccine (IIV4), and 23-valent pneumococcal polysaccharide vaccine (PPV23), particularly in different age groups, is still unknown.

**Methods:**

A phase 4, randomized, open-label, controlled trial was conducted in Beijing, China. 636 healthy adults were divided into two age groups (18-59 and ≥60 years) and randomized equally into three groups: CoronaVac and IIV4 followed by PPV23; CoronaVac and PPV23 followed by IIV4; or CoronaVac followed by IIV4 and PPV23, with a 28-day interval between vaccinations. Immunogenicity was evaluated by measuring antibody titers, and safety was monitored. ClinicalTrials.gov Identifier: NCT05298800.

**Results:**

Co-administration of a third dose of CoronaVac, IIV4, and PPV23 in any combination was safe. Among adults aged 18-59, co-administration with PPV23 maintained non-inferiority of antibody levels for CoronaVac and IIV4, despite a slight reduction in antibody responses. This reduction was not observed in participants ≥60 years. Furthermore, co-administration of IIV4 and PPV23 affected seroconversion rates for both vaccines.

**Conclusions:**

Co-administration of the third dose of SARS-CoV-2 inactivated vaccine with the influenza vaccine, followed by PPV23, may be optimal for adults aged 18-59. In adults ≥60, all vaccine combinations were immunogenic, suggesting a flexible vaccination approach. Since antibody measurements were taken 28 days post-vaccination, ongoing surveillance is essential to assess the longevity of the immune responses.

## Introduction

COVID-19, the disease caused by SARS-CoV-2, first emerged in humans in December 2019, and rapidly became a global pandemic. The accelerated development and deployment of vaccines against SARS-CoV-2 have helped to limit COVID-19-related severe disease, hospitalizations, and deaths (1, 2). Among the vaccines authorized for emergency use, inactivated SARS-CoV-2 vaccines have been widely employed and extensively administered in various countries, including China (3).

As new SARS-CoV-2 variants emerged, the importance of booster doses to sustain protective immunity and address waning antibody levels has become increasingly apparent (4, 5). Concurrently, other respiratory pathogens continue to pose significant public health and socioeconomic burdens. In China, influenza and *S. pneumoniae* are the leading viral and bacterial pathogens that cause acute respiratory diseases, contributing substantially to morbidity and mortality (6). Several city governments in China have made tremendous efforts to expand the availability of the influenza vaccine and 23-valent pneumococcal polysaccharide vaccine (PPV23) (7).

Given the overlapping vaccination needs and the potential stress on healthcare systems from administering multiple vaccines separately, exploring the feasibility and safety of concomitant COVID-19 booster doses with these vaccines is imperative (8). Clinical studies have shown that the co-administration of three COVID-19 vaccines (BNT162b2, ChAdOx1, and NVX-CoV2373) with seasonal influenza vaccines raised no safety concerns and confirmed the acceptable immunogenicity and reactogenicity of both vaccines (9, 10). Two phase 4 trials conducted in China showed that the co-administration of a first or second dose of inactivated SARS-CoV-2 vaccine with influenza or PPV23 vaccine did not raise any safety concerns (11, 12). Based on this evidence, the World Health Organization (WHO) promoted the co-administration of COVID-19 vaccines with inactivated influenza vaccines. However, as countries with widespread two-dose inactivated COVID-19 vaccination coverage shift focus to booster doses, there remains a knowledge gap concerning different combinations of booster shots with influenza and pneumococcal vaccines. Moreover, the immunogenicity, safety, and optimal vaccination strategies for co-administering inactivated SARS-CoV-2 booster doses with these vaccines, particularly across different age groups, are still unknown.

In this study, we conducted a clinical trial to assess the immunogenicity and safety of different combinations involving the booster dose of the inactivated SARS-CoV-2 vaccine (CoronaVac), the inactivated quadrivalent influenza vaccine (IIV4), and the PPV23 vaccine in adults aged 18-59 and those aged ≥60 years.

## Materials and methods

### Study design and participants

A single-center, open­label, randomized controlled trial was conducted in Beijing to assess the immunogenicity and safety of different combinations involving the 3rd booster dose of CoronaVac, IIV4, and PPV23. Healthy subjects aged ≥18 years and had received 2 doses of inactivated COVID-19 vaccine at least six months before the study’s commencement were enrolled. The main exclusion criteria included a history of SARS-CoV, SARS-CoV-2, or Middle East respiratory syndrome infection; receipt of any influenza vaccine within the preceding 6 months or any licensed vaccine; receipt of the pneumococcal vaccine within the last 5 years; axillary temperature exceeding 37.0°C; and history of allergy to any vaccine component. A comprehensive list of exclusion criteria is detailed in the protocol.

### Randomization and masking

Each eligible participant was assigned a random number based on the sequence of enrollment and then randomized into three groups: the first group received concomitant administration of SARS-CoV-2 and IIV4 followed by PPV23 (SARS-CoV-2+IIV4/PPV23); the second group received concomitant administration of SARS-CoV-2 and PPV23 followed by IIV4 (SARS-CoV-2+PPV23/IIV4); and the third group received separate administration of SARS-CoV-2 followed by concomitant administration of IIV4 and PPV23 (SARS-CoV-2/IIV4+PPV23). Each group is stratified into two subgroups by age (18–59 years vs ≥60 years, at a ratio of 1:1). The randomization was carried out according to a randomization list (block size=8) prepared by an independent statistician using SAS 9.4 (SAS Institute Inc., Cary, NC, USA). The random allocation was sealed using a numbered ‘‘scratch card”, group information covered by opaque material was not allowed to be scratched off unless randomization had been completed. Although participants and investigators were not masked to study group assignment, the laboratory staff, who were responsible for the sample testing, were masked to which vaccines were administered to each of the three groups.

### Study vaccines

The inactivated SARS-CoV-2 vaccine, CoronaVac, was developed by Sinovac Life Sciences Co., Ltd, and packed in vials with 0.5 ml each containing 600 SU/0.5 ml of SARS-CoV-2 antigen. IIV4 was produced by Sinovac Biotech Co., Ltd., which was formulated to contain 15 μg haemagglutinin/strain/0.5 ml, containing A/Victoria/2570/2019 (H1N1) pdm09, A/Cambodia/e0826360/2020 (H3N2), B/Washington/02/2019 (B/Victoria lineage), B/Phuket/3073/2013 (B/Yamagata lineage), which were recommended by WHO for Northern Hemisphere in 2021-2022 season. The PPV23 vaccine produced by Sinovac Biotech Co., Ltd. contains polysaccharides from pneumococcal serotypes 1, 2, 3, 4, 5, 6B, 7F, 8, 9 N, 9 V, 10A, 12F, 14, 15B, 17F,18C, 19A, 19F, 20, 22F, 23F, and 33F individually, each 0.5mL dose contains 25 μg of each polysaccharide. All vaccines were administered intramuscularly.

### Procedures

The SARS-CoV-2+IIV4/PPV23 group received one booster dose of CoronaVac and one dose of IIV4 on day 0, followed by one dose of PPV23 on day 28. The SARS-CoV-2 + PPV23/IIV4 group received one booster dose of CoronaVac and one dose of PPV23 on day 0, followed by one dose of IIV4 on day 28. The SARS-CoV-2/IIV4+PPV23 group received one booster dose of CoronaVac on day 0, followed by IIV4 and PPV23 on day 28. Three blood samples were collected from each participant: one before immunization on day 0, one before the second immunization on day 28, and one on day 56. The inactivated SARS-CoV-2 vaccine, CoronaVac, was provided by Sinovac Life Sciences Co., Ltd. IIV4 and PPV23 were produced by Sinovac Biotech Co., Ltd. All vaccines were administered intramuscularly.

### Immunogenicity assessment

Neutralizing antibodies (NAbs) against live Wuhan-Hu-1 strain were quantified using the micro cytopathogenic effect assay (13). SARS-CoV-2 neutralizing antibody seroconversion was defined as a change from seronegative to seropositive or a 4-fold titer increase if the participant was seropositive at baseline. The positive cutoff of the titer for neutralizing antibodies to live SARS-CoV-2 was 1/8 (13). The IIV4 antibody titers were detected by hemagglutinin inhibition (HI) assays (14). Seroconversion of IIV4 antibodies was defined as an HI antibody titer after vaccination of 1:40 or higher if<1:10 at baseline, or at least a 4-fold increase compared with baseline if the HI antibody titer was 1:10 or higher. The PPV23 IgG antibody concentration was determined using an in-house ELISA (15). IgG antibody seroconversion of 23 serotypes of *S. pneumoniae* was defined as a minimum two-fold increase in IgG antibody concentration compared with baseline.

### Safety assessment

Participants were monitored for immediate adverse events (AEs) within 30 minutes after each dose. AEs that occurred within 28 days after each dose were documented on diary cards. Within 7 days after each dose, participants were required to record any local AEs at the injection site and systemic AEs on diary cards. Solicited local adverse events included injection site pain, induration, swelling, erythema, rash, and pruritus. Solicited systemic adverse events included fever, cough, headache, acute hypersensitive reaction, diarrhea, nausea, myalgia, and fatigue. On Day 7, investigators conducted a face-to-face interview to ensure completeness and accuracy. Adverse events were monitored between Day 8 and 28 after each dose of the medication. The data was collected through participant reports and regular visits on Day 14 and Day 28. Grading of AEs was conducted following a scale provided by the National Medical Product Administration.

### Statistical analysis

The sample size was determined using NCSS-PASS software (version 11.0) based on the seroconversion rate of SARS-CoV-2 neutralizing antibodies 28 days after the third boost dose of CoronaVac in individuals aged 18 and above. We used a seroconversion rate of 95% to calculate the sample size. Ensuring a power of 80%, a one-sided alpha of 0.025, and a non-inferiority margin (Δ) of -10%, a minimum sample size of 153 participants per group would be necessary. Considering a potential 20% loss to follow-up, the sample size in each group was defined as 200. For the exploratory age subgroup analysis, participants were divided into two subgroups: those aged 18-59 and those aged 60 and above, maintaining a 1:1 ratio. Each group consisted of 100 adults aged 18-59 and 100 individuals aged 60 and above. For the immunological assessment, non-inferiority for the geometric mean titers (GMTs) or geometric mean concentrations (GMCs) ratio of the concomitant administration group is considered to be fulfilled when the lower boundary of 95% CI is 0.5 or higher compared with the separate administration group (11). Non-inferiority for the seroconversion rate of the concomitant administration group would be considered achieved if the lower bound of the two-sided 95% CI of the difference was > -10% compared with the separate administration group (11). The level of significance for the comparison was two-sided at 0.05. All statistical analyses were done using SAS version 9.4 or above.

## Results

### Participants

**Figure 1:**
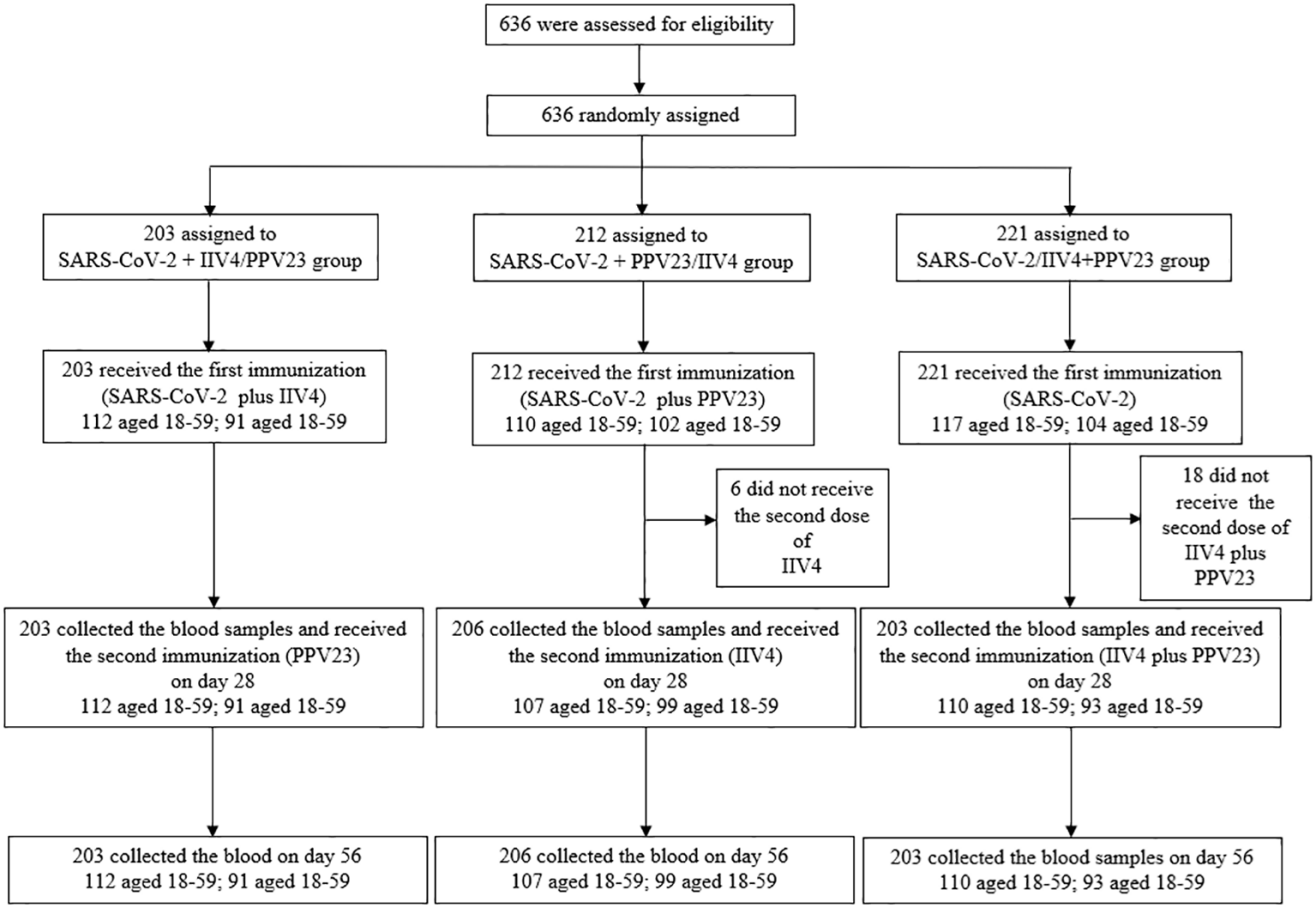
Trial profile. IIV4: quadrivalent split-virion inactivated influenza vaccine. PPV23: 23-valent pneumococcal polysaccharide vaccine. The dashed lines represent the 95% CI lower boundary of 0.5 for GMT ratios and -10% for the seroconversion rate differences.

From September 28 to November 28, 2020, 636 participants aged over 18 years were enrolled in this phase 4 trial, with 612 completing the study. These participants were randomly assigned to three groups: the SARS-CoV-2 + IIV4/PPV23 group (203 participants; 112 aged 18–59 years and 91 aged ≥60 years), the SARS-CoV-2 + PPV23/IIV4 group (206 participants; 107 aged 18–59 years and 99 aged ≥60 years), and the SARS-CoV-2/IIV4+PPV23 group (203 participants; 110 aged 18–59 years and 93 aged ≥60 years) (**Figure 1**). The participants in the three groups were well-balanced in demography, including mean age, sex, ethnicity, height, and weight. Most participants (576/612, 94.12%) were of Han Chinese ethnicity, and 36 (5.88%) were of non-Han Chinese ethnicity (**Table 1**).

**Table 1:** Characteristics of the participants in the study.

Characteristics	SARS-CoV-2 + IIV4/PPV23 group	SARS-CoV-2 + PPV23/IIV4 group	SARS-CoV-2/IIV4+PPV23 group
	(N=203)	(N=206)	(N=203)
Age (years)			
Mean (SD)	50.77	50.75	51.11
Median	53	55	53
Min, Max	23,74	20,84	22,86
Sex, n (%)			
Male	110(54.19)	122(59.22)	125(61.58)
Female	93(45.81)	84(40.78)	78(38.42)
Ethnic [n (%)]			
Han	192(94.58)	192(93.20)	192(94.58)
Others	11(5.42)	14(6.80)	11(5.42)
Height (cm)			
Mean (SD)	167.1(8.003)	168(7.941)	167.3(7.73)
Median	168	169	168
Min, Max	148,187	145,193	150,189
Weight (kg)			
Mean (SD)	69.89(11.08)	71.63(13.8)	70.14(11.8)
Median	70	70	70
Min, Max	40,110	47.5,130	48,101
BMI, kg/m2, median (IQR)			
Male	25.21	25.81	25.18
Female	24.66	24.47	24.61

### Immunogenicity

#### Response to CoronaVac

At baseline, the GMTs against the Wuhan-Hu-1 strain did not reach the positive threshold of 1:8, with no significant differences in baseline GMTs and seropositivity rates among the three groups (**Table 2**).

**Table 2:** Antibody responses to SARS-CoV-2 strains.

	SARS-CoV-2 + IIV4/PPV23 group	SARS-CoV-2 + PPV23/IIV4 group	SARS-CoV-2/IIV4+PPV23 group	P value		95% CI		Non-inferiority^*^	
	Group 1	Group 2	Group 3	Group 1&3	Group 2&3	Group 1&3	Group 2&3	Group 1&3	Group 2&3
Seropositivity-BI [n (%)]	35.29	36.54	34.63	0.918	0.758				
GMT-BI	5.39	5.72	5.51	0.505	0.999				
Seropositivity [n (%)]	98.04	100.00	98.54	0.724	0.121				
Seroconversion [n (%)]	93.14	95.67	94.63	0.544	0.654	-1.49(-6.13,3.15)	1.04(-3.1,5.18)	Yes	Yes
GMT	114.70	120.30	161.80	0.012	0.033	0.71(0.57,0.88)	0.74(0.6,0.92)	Yes	Yes
Aged 18-59									
GMT	106.4	112.9	171.2	<0.01	0.01	0.62(0.51,0.83)	0.66(0.50,0.88)	Yes	Yes
Seroconversion [n (%)]	92.86	95.37	92.73	1.000	0.524	0.13(-6.67,6.93)	2.64(-3.63,8.91)	Yes	Yes
Aged ≥60									
GMT	125.70	128.90	151.50	0.182	0.189	0.83(0.59,1.16)	0.85(0.62,1.17)	Yes	Yes
Seroconversion [n (%)]	93.48	96.84	95.47	0.325	1.000	-3.36(-9.51,2.79)	-0.84(-6.05,4.37)	Yes	Yes

*Non-inferiority for the GMT ratio is considered to be fulfilled when the lower boundary of 95% CI is 0.5 or higher. Non-inferiority for the seroconversion rate would be considered as achieved if the lower bound of the two-sided 95% CI of the difference was > -10%.BI: before injection; GMT, geometric mean titre; Blood samples were collected at 28 days after each group received the inactivated SARS-CoV-2 vaccine. For antibody responses to SARS-CoV-2, seropositivity criteria: The neutralizing antibody titer is ≥1:8; seroconversion criteria: the neutralizing antibody titer is < 1:8 before immunization and ≥ 1:8 after immunization; or the neutralizing antibody titer is ≥ 1:8 before immunization and increases by 4 times and above after immunization.

28 days after the booster dose of CoronaVac, the NAbs in the CoronaVac alone group showed significantly higher GMT compared to the group that received CoronaVac plus IIV4 (161.8 vs 114.7, p=0.012) or PPV23 (161.8 vs 120.3, p=0.033) against the Wuhan-Hu-1 strain (**Table 2**). Non-inferiority for the GMTs and seroconversion rates were achieved when CoronaVac was co-administered with the IIV4 or PPV23 (**Table 2**).

**Figure 2:**
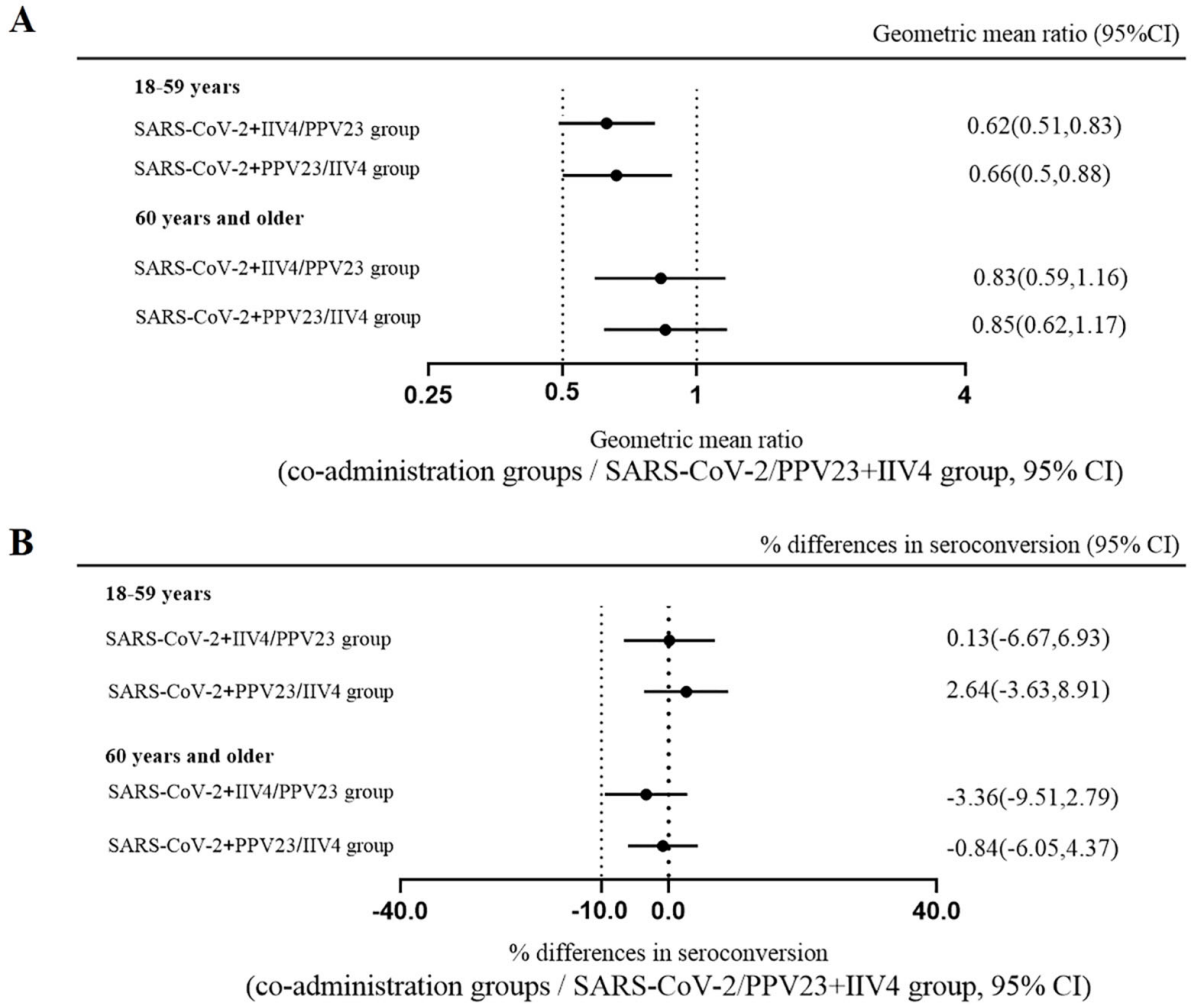
Non-inferiority for SARS-CoV-2 neutralizing antibody geometric mean titer (GMT) ratios and seroconversion rate of co-administration versus separate administration. Blood samples were collected 28 days after each group received CoronaVac. Neutralizing antibodies against live Wuhan-Hu-1 strain were quantified using the micro cytopathogenic effect assay. The GMT ratios **(A)** and differences in seroconversion rates **(B)** were compared between the co-administration groups and the SARS-CoV-2/PPV23+IIV4 group. The dashed lines represent the 95% CI lower boundary of 0.5 for GMT ratios and -10% for the seroconversion rate differences.

In participants aged 18-59, the GMT against the Wuhan-Hu-1 strain was significantly higher in the CoronaVac alone group than the group combined with the IIV4 (106.4 vs 171.2, *p*<0.01) or PPV23 (112.9 vs 171.2, *p*=0.01) (**Table 1**). Non-inferiority for NAbs levels and seroconversion rate were achieved when CoronaVac was combined with IIV4 or PPV23 (**Figure 2A**). In participants aged 60 and above, there was no significant difference in NAbs levels between the concomitant groups and the CoronaVac alone group (**Table 1**). Non-inferiority for GMTs and seroconversion rates was also achieved when CoronaVac was combined with IIV4 or PPV23 (**Figure 2B**). These results suggest that while there was a decrease in antibody responses to the third dose of CoronaVac, co-administration with IIV4 or PPV23 did not affect the non-inferiority of neutralizing antibody levels and seroconversion rates for CoronaVac in individuals aged 18-59. Moreover, no negative impact was observed in people aged 60 and above.

#### Response to IIV4

At baseline, GMTs and seropositivity rates for antibodies against four influenza strains were similar across all groups, except for the B/Yamagata strain, where differences were observed between the IIV4 plus PPV23 group and the IIV4 alone group (**Table 3**).

**Table 3:** Antibody responses to four types of influenza strains.

	SARS-CoV-2 + IIV4/PPV23 group	SARS-CoV-2 + PPV23/IIV4 group	SARS-CoV-2/IIV4+PPV23 group	P value		95% CI		Non-inferiority^*^	
	Group 1	Group 2	Group 3	Group 1&2	Group 3&2	Group 1&2	Group 3&2	Group 1&2	Group 3&2
A/H1N1									
Seropositivity-BI [n (%)]	29.32	25.76	32.46	0.432	0.145				
GMT-BI	17.23	16.56	17.81	0.732	0.509				
Seropositivity [n (%)]	96.86	94.95	95.29	0.343	0.877				
Seroconversion [n (%)]	87.43	90.91	85.34	0.270	0.089	-3.47(-9.65,2.7)	-5.57(-11.99,0.85)	Yes	No
GMT	475.30	488.80	307.50	0.843	0.001	0.97(0.74,1.28)	0.63(0.48,0.83)	Yes	No
A/H3N2									
Seropositivity-BI [n (%)]	36.65	35.86	43.46	0.871	0.126				
GMT-BI	26.45	26.37	28.65	0.967	0.174				
Seropositivity [n (%)]	98.95	98.48	97.91	0.682	0.668				
Seroconversion [n (%)]	86.91	80.30	65.97	0.079	0.001	6.61(-0.71,13.93)	-14.33(-23.04,-5.63)	Yes	No
GMT	223.40	177.70	129.60	0.028	0.001	1.26(1.03,1.54)	0.73(0.6,0.88)	Yes	Yes
B/Victoria									
Seropositivity-BI [n (%)]	35.08	41.92	46.60	0.166	0.353				
GMT-BI	24.96	28.38	30.36	0.133	0.426				
Seropositivity [n (%)]	93.19	95.96	94.24	0.228	0.432				
Seroconversion [n (%)]	67.54	69.19	56.54	0.726	0.010	-1.65(-10.9,7.59)	-12.65(-22.18,-3.12)	No	No
GMT	141.90	148.10	118.00	0.697	0.040	0.96(0.77,1.19)	0.8(0.64,0.99)	Yes	Yes
B/Yamagata									
Seropositivity-BI [n (%)]	56.02	54.04	59.69	0.695	0.261				
GMT-BI	37.47	36.01	40.58	0.624	0.148				
Seropositivity [n (%)]	97.38	96.46	96.86	0.601	0.829				
Seroconversion [n (%)]	68.59	67.68	61.78	0.847	0.224	0.91(-8.35,10.17)	-5.9(-15.38,3.59)	Yes	No
GMT	196.10	177.70	149.90	0.364	0.905	1.1(0.89,1.36)	0.84(0.7,1.02)	Yes	Yes

*Non-inferiority for the GMT ratio is considered to be fulfilled when the lower boundary of 95% CI is 0.5 or higher. Non-inferiority for the seroconversion rate would be considered as achieved if the lower bound of the two-sided 95% CI of the difference was > -10%. BI, before injection; GMT, geometric mean titre; Blood samples were collected at 28 days after each group received the influenza vaccine. Seropositivity criteria: The protective level of HI antibody titer against certain antigen is ≥ 1:40; seroconversion criteria: the HI antibody titer is<1:10 before vaccination and ≥1:40 after vaccination; the HI antibody titer is ≥ 1:10 before vaccination and increased by four times after vaccination.

On day 28 after IIV4 vaccination, the IIV4 plus CoronaVac group showed non-inferiority for GMTs and seroconversion rates against all four strains, except for the B/Victoria seroconversion rate, which showed a non-inferiority margin of -1.65% (95% CI, -10.9, 7.59) (**Table 3**). In contrast, when IIV4 was co-administered with PPV23, GMTs against three of the four strains met non-inferiority criteria, but none of the seroconversion rates met non-inferiority criteria (**Table 3**).

**Figure 3:**
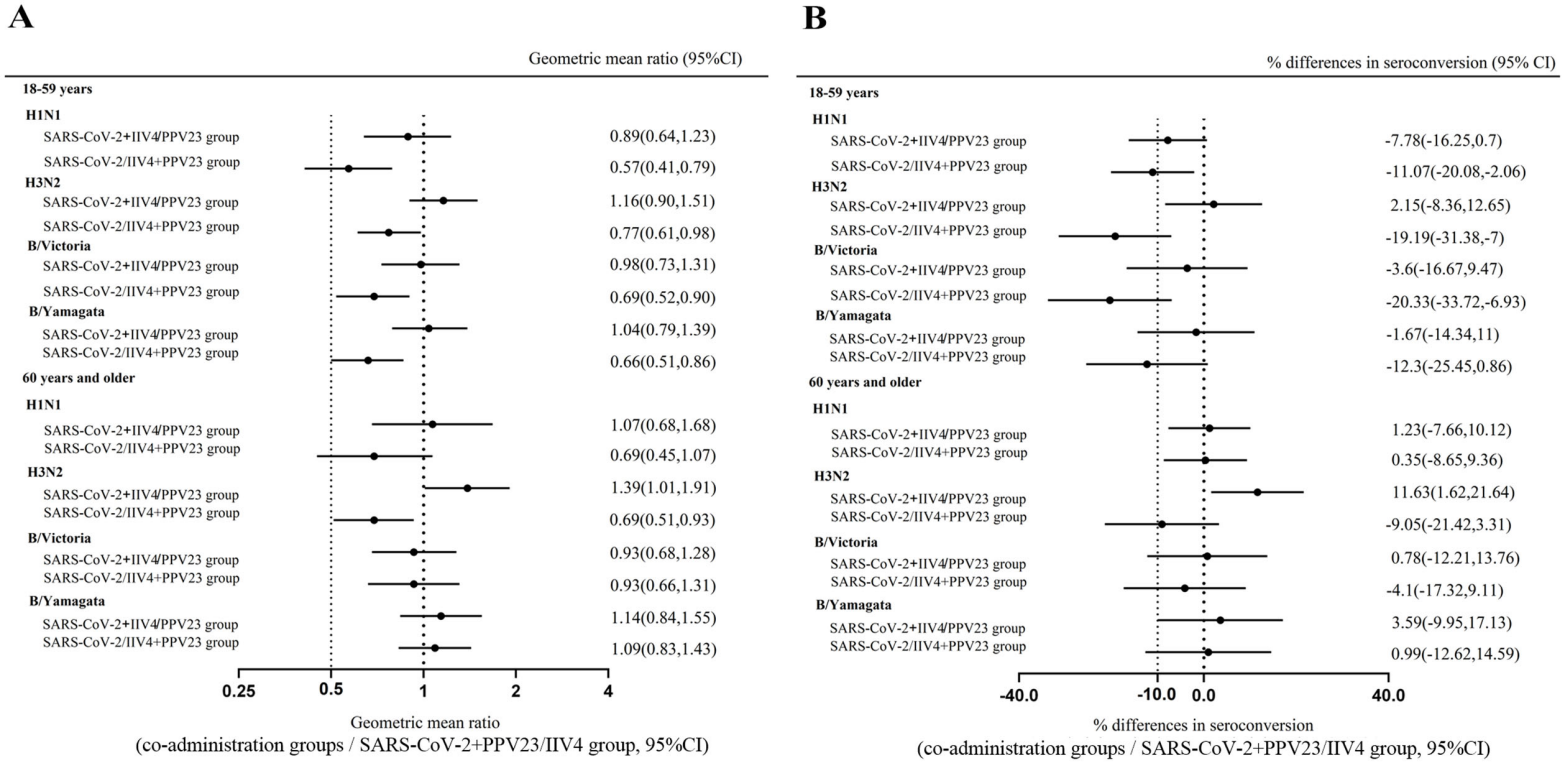
Non-inferiority for influenza haemagglutination inhibition geometric mean titer (GMT) ratios and seroconversion rate of co-administration versus separate administration. Blood samples were collected 28 days after each group received inactivated quadrivalent influenza vaccine. The antibody titers against the four strains included in the vaccine were detected by hemagglutinin inhibition assays. The GMT ratios **(A)** and differences in seroconversion rates **(B)** were compared between the co-administration groups and the SARS-CoV-2+PPV23/IIV4 group. The dashed lines represent the 95% CI lower boundary of 0.5 for GMT ratios and -10% for the seroconversion rate differences.

In the 18-59 age group, no differences in GMTs against four influenza strains were noted between the IIV4 plus CoronaVac group and the IIV4 alone group. However, GMTs were significantly lower in the IIV4 plus PPV23 group compared to the IIV4 alone group. In the ≥60 years age group, no significant differences in GMTs were observed between the concurrent groups and the IIV4 alone group, except for a lower GMT against A/H3N2 in the IIV4 plus PPV23 group. In both age groups, co-administration of IIV4 with either CoronaVac or PPV23 demonstrated non-inferiority concerning GMTs against the four influenza strains, except for the A/H1N1 strain when combined with PPV23 (**Figure 3A**). Besides, in the 18-59 age group, the non-inferiority for the seroconversion rates against four strains in two concomitant groups was only achieved when IIV4 was combined with CoronaVac against A/H3N2 (2.15% [95% CI, -8.36, 12.65]) (**Figure 3B**). Moreover, in the ≥60 years age group, three of the four strains met the non-inferiority criteria for seroconversion rate when IIV4 was co-administered with CoronaVac, whereas only one of the four strains met the non-inferiority criteria for seroconversion rate when IIV4 was co-administered with PPV23 (2.15% [95% CI, -8.36, 12.65]) (**Figure 3B**). These findings indicate that concurrent administration of PPV23 slightly affects the non-inferiority of GMTs for IIV4, with a reduction in antibody responses to IIV4 observed in the 18-59 age group. Moreover, co-administration of PPV23 impacts the seroconversion rates of IIV4 across both age groups.

#### Response to PPV23

Before vaccination, despite the significant variability in baseline IgG geometric mean concentrations (GMCs) for different serotypes, no significant differences were observed among the three groups for 19 out of 23 pneumococcal serotypes, except serotypes 2, 7, and 12F (**Table 4**).

**Table 4:** Antibody responses to 23 pneumococcal serotypes.

Serotype		SARS-CoV-2 + IIV4/PPV23 group	SARS-CoV-2 + PPV23/IIV4 group	SARS-CoV-2/IIV4+PPV23 group	P value		95% CI		Non-inferiority^*^	
		Group 1	Group 2	Group 3	Group 2&1	Group 3&1	Group 2&1	Group 3&1	Group 2&1	Group 3&1
**1**	Seroconversion [n (%)]	92.05	90.10	90.54	0.590	0.694	-1.95(-7.69,3.79)	-1.5(-7.69,4.68)	Yes	Yes
	GMC	10.21	9.47	8.16	0.614	0.025	0.93(0.7,1.23)	0.8(0.61,1.05)	Yes	Yes
**2**	Seroconversion [n (%)]	91.19	93.60	89.30	0.448	0.605	2.4(-2.82,7.63)	-1.89(-7.85,4.08)	Yes	Yes
	GMC	14.75	15.76	12.67	0.472	0.275	1.07(0.82,1.4)	0.86(0.66,1.12)	Yes	Yes
**3**	Seroconversion [n (%)]	61.50	65.52	58.96	0.461	0.667	4.02(-5.54,13.58)	-2.54(-12.66,7.58)	Yes	No
	GMC	0.97	1.55	0.95	<0.001	0.858	1.6(1.29,1.99)	0.98(0.79,1.21)	Yes	Yes
**4**	Seroconversion [n (%)]	86.54	83.59	80.61	0.461	0.667	-2.95(-10.41,4.52)	-5.93(-14,2.14)	No	No
	GMC	3.08	3.47	2.35	0.045	0.163	1.13(0.87,1.46)	0.76(0.6,0.98)	Yes	Yes
**5**	Seroconversion [n (%)]	84.97	81.28	83.96	0.350	0.887	-3.69(-11.06,3.67)	-1.02(-8.3,6.27)	No	Yes
	GMC	4.71	5.03	4.75	0.201	0.521	1.07(0.83,1.38)	1.01(0.79,1.3)	Yes	Yes
**6B**	Seroconversion [n (%)]	80.00	74.36	71.43	0.222	0.068	-5.64(-14.05,2.77)	-8.57(-17.31,0.16)	No	No
	GMC	7.42	6.87	6.99	0.766	0.435	0.93(0.73,1.17)	0.94(0.74,1.2)	Yes	Yes
**7**	Seroconversion [n (%)]	84.30	95.57	88.77	<0.001	0.219	11.26(5.13,17.39)	4.47(-2.61,11.54)	Yes	Yes
	GMC	9.99	12.31	9.49	0.268	0.287	1.23(0.97,1.57)	0.95(0.75,1.21)	Yes	Yes
**8**	Seroconversion [n (%)]	86.63	86.87	81.29	1.000	0.194	0.24(-6.54,7.01)	-5.34(-12.96,2.27)	Yes	No
	GMC	14.12	16.04	13.06	0.012	0.626	1.14(0.93,1.38)	0.92(0.77,1.11)	Yes	Yes
**9N**	Seroconversion [n (%)]	91.19	94.09	89.84	0.335	0.727	2.9(-2.25,8.05)	-1.35(-7.25,4.54)	Yes	Yes
	GMC	15.52	16.96	13.21	0.845	0.140	1.09(0.87,1.37)	0.85(0.67,1.08)	Yes	Yes
**9V**	Seroconversion [n (%)]	85.49	82.76	82.12	0.494	0.400	-2.73(-9.92,4.46)	-3.37(-10.87,4.13)	Yes	No
	GMC	9.29	9.37	8.64	0.343	0.282	1.01(0.81,1.25)	0.93(0.75,1.15)	Yes	Yes
**10A**	Seroconversion [n (%)]	88.46	86.73	89.39	0.746	0.862	-1.73(-8.63,5.18)	0.92(-5.82,7.67)	Yes	Yes
	GMC	15.04	13.57	12.92	0.545	0.252	0.9(0.67,1.21)	0.86(0.64,1.16)	Yes	Yes
**11A**	Seroconversion [n (%)]	79.27	81.28	75.00	0.705	0.369	2.01(-5.84,9.85)	-4.27(-13.16,4.61)	Yes	No
	GMC	8.43	9.96	7.60	0.037	0.105	1.18(0.95,1.47)	0.9(0.73,1.12)	Yes	Yes
**12F**	Seroconversion [n (%)]	72.04	71.43	71.12	0.911	0.909	-0.61(-9.57,8.34)	-0.92(-10.07,8.23)	Yes	No
	GMC	3.07	3.81	3.13	0.204	0.193	1.24(0.99,1.55)	1.02(0.82,1.26)	Yes	Yes
**14**	Seroconversion [n (%)]	75.98	77.11	74.33	0.809	0.719	1.14(-7.4,9.67)	-1.65(-10.5,7.21)	Yes	No
	GMC	14.44	16.36	14.34	0.669	0.325	1.13(0.84,1.53)	1.04(0.77,1.42)	Yes	Yes
**15B**	Seroconversion [n (%)]	86.02	83.08	87.70	0.480	0.649	-2.94(-10.19,4.3)	1.68(-5.18,8.53)	No	Yes
	GMC	22.93	19.06	19.29	<0.001	0.034	0.83(0.65,1.06)	0.84(0.67,1.05)	Yes	Yes
**17F**	Seroconversion [n (%)]	87.01	90.61	88.77	0.316	0.632	3.6(-2.92,10.13)	1.76(-4.95,8.47)	Yes	Yes
	GMC	8.19	10.90	8.12	0.873	0.225	1.33(1.02,1.73)	0.99(0.76,1.3)	Yes	Yes
**18C**	Seroconversion [n (%)]	83.33	90.91	84.97	0.031	0.773	7.58(0.82,14.33)	1.64(-5.91,9.19)	Yes	Yes
	GMC	9.63	9.43	9.22	0.790	0.571	0.98(0.78,1.22)	0.96(0.78,1.18)	Yes	Yes
**19A**	Seroconversion [n (%)]	79.89	85.20	75.94	0.178	0.383	5.31(-2.32,12.94)	-3.96(-12.39,4.48)	Yes	No
	GMC	8.02	9.29	6.69	0.123	0.512	1.16(0.83,1.61)	0.83(0.6,1.15)	Yes	Yes
**19F**	Seroconversion [n (%)]	79.33	76.29	74.33	0.534	0.268	-3.04(-11.47,5.39)	-5.00(-13.62,3.63)	No	No
	GMC	9.50	9.76	7.93	0.539	0.022	1.03(0.81,1.3)	0.83(0.67,1.04)	Yes	Yes
**20A**	Seroconversion [n (%)]	69.02	64.29	62.01	0.384	0.185	-4.74(-14.2,4.73)	-7.01(-16.77,2.75)	No	No
	GMC	11.22	9.07	8.99	0.258	0.020	0.81(0.63,1.04)	0.8(0.63,1.02)	Yes	Yes
**22F**	Seroconversion [n (%)]	79.88	86.90	81.62	0.104	0.770	7.03(-0.95,15.01)	1.74(-7.21,10.69)	Yes	Yes
	GMC	4.51	6.23	5.34	0.215	0.895	1.38(1.05,1.81)	1.18(0.9,1.55)	Yes	Yes
**23F**	Seroconversion [n (%)]	80.54	76.29	80.75	0.322	1.000	-4.25(-12.52,4.02)	0.21(-7.82,8.24)	No	Yes
	GMC	5.58	5.38	4.66	0.773	0.109	0.96(0.74,1.26)	0.84(0.65,1.08)	Yes	Yes
**33F**	Seroconversion [n (%)]	91.85	93.58	91.44	0.554	1.000	1.74(-3.55,7.02)	-0.4(-6.03,5.23)	Yes	Yes
	GMC	26.94	28.18	23.46	0.897	0.077	1.05(0.83,1.32)	0.87(0.69,1.1)	Yes	Yes

*Non-inferiority for the GMT ratio is considered to be fulfilled when the lower boundary of 95% CI is 0.5 or higher. Non-inferiority for the seroconversion rate would be considered as achieved if the lower bound of the two-sided 95% CI of the difference was > -10%. Blood samples were collected at 28 days after each group received the PPV23. IgG antibody seroconversion of 23 serotypes of S pneumoniae was defined as a minimum two-fold increase in the post-vaccination IgG antibody concentration compared with baseline.

On day 28 after PPV23 vaccination, non-inferiority of the GMCs was achieved for all 23 pneumococcal serotypes when PPV23 was concomitantly administered with IIV4 or CoronaVac (**Table 4**). Additionally, non-inferior seroconversion rates were achieved for 12 or 16 pneumococcal serotypes when PPV23 was co-administered with IIV4 or CoronaVac, respectively (**Table 4**).

**Figure 4:**
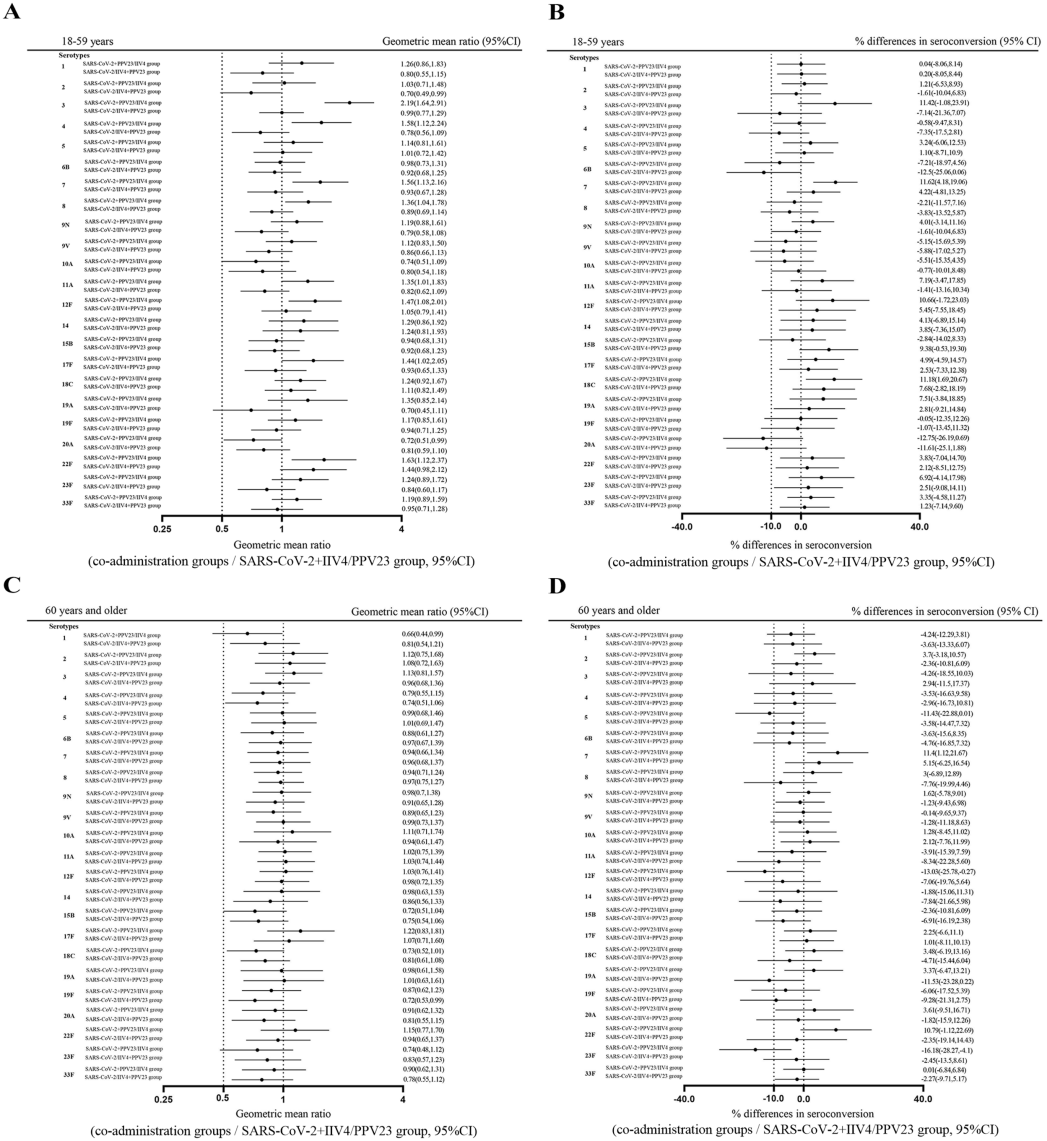
Non-inferiority for 23-valent pneumococcal polysaccharide vaccine (PPV23) IgG geometric mean concentrations (GMC) ratios and seroconversion rate of co-administration versus separate administration. Blood samples were collected 28 days after each group received the PPV23 vaccine. The PPV23 IgG antibody concentration was determined using an in-house ELISA. The PPV23 IgG GMT ratios of the 18-59 age group **(A)** and ≥60 age group **(C)** were compared between the co-administration groups and the SARS-CoV-2+IIV4/PPV23 group. The differences in seroconversion rates of the 18-59 age group **(B)** and ≥60 age group **(D)** were compared between the co-administration groups and the separate administration group. The dashed lines represent the 95% CI lower boundary of 0.5 for GMT ratios and -10% for the seroconversion rate differences.

In the 18-59 age group, co-administration of PPV23 with IIV4 led to a decrease in IgG GMCs for 18 pneumococcal serotypes compared to PPV23 alone. Conversely, when PPV23 was combined with CoronaVac, higher GMCs were noted for 19 pneumococcal serotypes, with significant increases observed in seven serotypes, compared to those who received PPV23 alone. Non-inferiority of GMCs was achieved for 23 or 21 pneumococcal serotypes when PPV23 was concurrently administered with CoronaVac or IIV4 (**Figure 4A**). Non-inferior seroconversion rates were achieved for 12 and 16 pneumococcal serotypes when PPV23 was co-administered with IIV4 and CoronaVac, respectively (**Figure 4B**). In the ≥60 years age group, non-inferiority for the GMCs was achieved for 23 or 21 pneumococcal serotypes when PPV23 was co-administered with IIV4 or CoronaVac, respectively (**Figure 4C**). Non-inferiority of seroconversion rates was achieved for 5 or 11 pneumococcal serotypes when PPV23 was co-administered with IIV4 or CoronaVac (**Figure 4D**). These results suggest that concurrent administration of either IIV4 or CoronaVac has a marginal impact on the non-inferiority of GMCs to PPV23; however, concomitant administration affects the seroconversion rates of PPV23.

### Safety

All vaccines administered during the study were well tolerated, and no severe adverse events or deaths were observed in any participant. Overall, 612 participants reported 90 vaccine-related adverse reactions during the 0-56 days after the first dose, with a rate of 14.78% in the SARS-CoV-2+IIV4/PPV23 group, 13.59% in the SARS-CoV-2+PPV23/IIV4 group, and 15.76% in the SARS-CoV-2/IIV4+PPV23 group (**Table 5**). The occurrence rates of solicited and vaccination-related unsolicited AEs were similar among the three groups. The most frequently reported adverse reaction was pain at the injection site, which occurred in more than 10% of the participants in all three groups. Most of the reported AEs were mild or moderate. Only one case of grade 3 (severe) cough was observed in the SARS-CoV-2+IIV4/PPV23 group. Besides, both the occurrence rates of solicited and vaccination-related unsolicited AEs were similar between the two age subgroups in each vaccination group. No serious vaccine-related adverse events were observed.

**Table 5:** Reported vaccine-related adverse events by the groups among participants.

Adverse events	SARS-CoV-2 + IIV4/PPV23 group N=203	SARS-CoV-2 + PPV23/IIV4 group N=206	SARS-CoV-2/IIV4+PPV23 group N=203	P value
	n (%)	n (%)	n (%)	
**TOTAL**	30(14.78)	28(13.59)	32(15.76)	0.834
**Solicited adverse reactions**	29(14.29)	27(13.11)	29(14.29)	0.925
**Systematic**	8(3.94)	7(3.40)	11(5.42)	0.594
Fever	3(1.48)	2(0.97)	1(0.49)	0.707
Cough	1(0.49)	2(0.97)	3(1.48)	0.707
Headache	2(0.99)	0(0.00)	5(2.46)	0.038
Nausea	2(0.99)	0(0.00)	1(0.49)	0.329
Fatigue	4(1.97)	5(2.43)	5(2.46)	1.000
Myalgia	2(0.99)	2(0.97)	7(3.45)	0.116
Diarrhea	0(0.00)	1(0.49)	2(0.99)	0.551
Myalgia	0(0.00)	0(0.00)	2(0.99)	0.219
**Local**	24(11.82)	24(11.65)	26(12.81)	0.951
Pain	24(11.82)	21(10.19)	24(11.82)	0.829
Rash	0(0.00)	1(0.49)	0(0.00)	1.000
Pain	0(0.00)	3(1.46)	1(0.49)	0.331
Induration	2(0.99)	9(4.37)	6(2.96)	0.111
**Unsolicited adverse reactions**	9(4.43)	5(2.43)	10(4.93)	0.360
**Systematic**	0(0.00)	0(0.00)	1(0.49)	0.663
Fatigue	0(0.00)	0(0.00)	1(0.49)	0.663
Myalgia	0(0.00)	0(0.00)	1(0.49)	0.663
**Local**	0(0.00)	1(0.49)	0(0.00)	1.000
Pain	0(0.00)	1(0.49)	0(0.00)	1.000
**Others**	9(4.43)	4(1.94)	9(4.43)	0.305

## Discussion

Our findings showed that the coadministration of CoronaVac, IIV4, and PPV23 in any combination raised no safety concerns in adults aged ≥18 years. It is noteworthy that co-administration of IIV4 or PPV23 with a third dose of CoronaVac, administered 28 days later, did not compromise the non-inferiority of neutralizing antibody levels and seroconversion rates of CoronaVac in adults aged 18 years and older. However, it slightly reduced antibody responses to CoronaVac within the 18-59 age group. Additionally, our findings indicated that while co-administration of PPV23 had a minimal effect on the non-inferiority of GMTs for IIV4 at 28 days post-PPV23 vaccination, it impacted the antibody responses to IIV4 in the 18-59 age group. Besides, the coadministration of IIV4 and PPV23 affected seroconversion rates for both vaccines.

Previous trials have mainly focused on the concomitant administration of the first dose of COVID-19 vaccines, with most studies showing no significant differences in reactogenicity between concomitant and separate vaccinations (9–12). However, a phase 3 trial revealed a significant decrease in anti-spike protein IgG ELISA units when NVX-CoV2373 (protein subunit vaccine) was co-administered with the influenza vaccine, failing to meet the non-inferiority of GMTs, indicating immune interference in those aged 18-64 years (10). Further analysis showed that higher anti-spike protein IgG ELISA units were reached in participants with serological evidence of previous COVID-19 infection. Therefore, Toback and colleagues have hypothesized that the pre-existing T-cell and B-cell populations with memory for the SARS-CoV-2 spike protein may reduce the negative effects of immune interference (10). This finding indicates that concomitant administration might impact priming but not subsequent responses, implying that it might be optimal to co-administer an influenza vaccine with second or later doses of the COVID-19 vaccine (9). In contrast, a study by Wang et al. found that participants vaccinated with IIV4 and the second dose of CoronaVac exhibited a significantly lower seroconversion rate and GMTs against SARS-CoV-2 compared to those concurrently vaccinated with the first dose or CoronaVac alone in the 18-59 age group (11). Consistent with these findings, our study revealed a decrease in antibody responses to the third dose of CoronaVac when co-administered with either IIV4 or PPV23 among individuals aged 18-59. However, such a negative impact was not observed in people aged 60 and above. These results suggest that for less immunogenic vaccines, such as protein subunit and inactivated vaccines, concurrent immunization might affect both priming and subsequent immune responses, specifically for adults aged 18-59 years (16). This finding should be assessed further as it has important implications for public health vaccination strategies.

In this study, we also observed that co-administration of PPV23 impacted the antibody responses to IIV4 and CoronaVac in the 18-59 age group, but not in aged 60 years and above. This disparity could be due to immunosenescence, where the immune function’s natural decline with age is more pronounced in older adults, potentially reducing the impact of PPV23 on antibody levels (17) (18). Additionally, younger individuals, who typically have stronger antigen-presenting cell function, may exhibit more complex immune responses to vaccine co-administration, resulting in observed reductions in antibody levels (19). Conversely, the decline in antigen-presenting cell function in older adults might lessen interference from PPV23, leading to more stable immune responses. Furthermore, the composition of PPV23 itself could contribute to this discrepancy. PPV23 contains a mixture of polysaccharide antigens derived from 23 pneumococcal serotypes. These antigens may interact differently with the immune system depending on age, potentially leading to variations in antibody responses (20). These findings underscore the need for further investigation into age-related differences in immune responses to optimize vaccination strategies across different age groups.

Consistent with previous research, our study demonstrated that the co-administration of IIV4 with PPV23 minimally impacted the non-inferiority of antibody titers for both vaccines (21, 22). However, this combination reduced the seroconversion rates for both vaccines. For the influenza vaccine, besides the immune interference from PPV23 mentioned earlier, the PPV23 plus IIV4 group exhibited the highest baseline antibody levels and seropositivity rates for all four influenza strains among the three vaccine groups, potentially influencing the seroconversion rates. Regarding PPV23, further analysis revealed that over half of the participants had baseline IgG levels for 15 out of 23 serotypes above 1.3 µg/mL (the World Health Organization’s defined threshold for protection against invasive pneumococcal disease) (23), suggesting that these individuals may have experienced repeated S. pneumoniae infections, which could have influenced the seroconversion rates. Furthermore, co-administration of IIV4 with PPV23 may have led to antigen overload, potentially complicating the immune response and contributing to the reduced seroconversion rates. Interestingly, our study also demonstrated that in the 18-59 age group, co-administration of PPV23 with CoronaVac resulted in higher GMCs for 19 pneumococcal serotypes, with significant increases observed for seven serotypes compared to PPV23 alone. However, the specific mechanism behind these findings remains unclear due to limited research on the immune response pathway and mechanism of the COVID-19 inactivated vaccine.

An advantage of inactivated COVID-19 vaccines is that, in addition to S protein, they also contain additional conserved SARSCoV-2 antigens (24). This comprehensive antigen exposure results in a diverse T-cell response targeting conserved epitopes across the S, N, and E proteins, providing cross-reactivity between the ancestral virus and emerging variants of concern (VOCs) (25). The T-cells target less antigenically changeable internal structural viral proteins, making inactivated vaccines less vulnerable to antibody escape mutations in VOCs (26). Consequently, inactivated COVID-19 vaccines are expected to be effective against both ancestral and variant SARS-CoV-2. Therefore, with the ongoing emergence of new COVID-19 variants, it is essential to explore the possibility of administering inactivated SARS-CoV-2 vaccines alongside other important vaccines such as those for influenza and pneumococcal disease.

Our study has several limitations. First, antibody measurements were conducted 28 days after each vaccination; therefore, there is a lack of understanding regarding long-term safety and sustained immunogenic responses. More follow-up is necessary. Second, the T-cell responses were not evaluated. Considering the recognized importance of cellular immunity in protecting against natural SARS-CoV-2 infection and the independent behavior of vaccine-induced cellular responses to neutralizing antibodies, further studies on T-cell responses during concurrent administration are needed (27). Finally, these findings are primarily based on data collected in China from almost a single ethnic group; thus, validating them across diverse ethnicities and countries is important for broader applicability.

In summary, this study identified that co-administration of CoronaVac, IIV4, and PPV23 in any combination was safe. For the 18-59 age group, co-administration of the third dose of inactivated SARS-CoV-2 vaccine with the influenza vaccine, followed by PPV23, could potentially optimize the immune response and might be a viable immunization strategy. In adults over 60, all combination of these vaccines maintains immunogenicity, offering flexibility in vaccination strategies. These findings are instrumental in crafting tailored vaccination strategies for different age demographics. However, more research is needed to help guide national immunization policies on this important issue.

## Data Availability

The original contributions presented in the study are included in the article/**Supplementary Material**. Further inquiries can be directed to the corresponding authors.
