# Supplementary material for: Immunogenicity and safety of different combinations involving a third booster dose of SARS-CoV-2 inactivated vaccine, inactivated quadrivalent influenza vaccine, and 23-valent pneumococcal polysaccharide vaccine in adults aged ≥60 years: a phase 4, randomized, open-label study
Source: Front Immunol. 2024 Aug 20;15:1437267. doi: 10.3389/fimmu.2024.1437267 (PMC11368774; doi:10.3389/fimmu.2024.1437267)
Supplement: Supplementary file 1 [file DataSheet1.docx]

**Supplementary material**


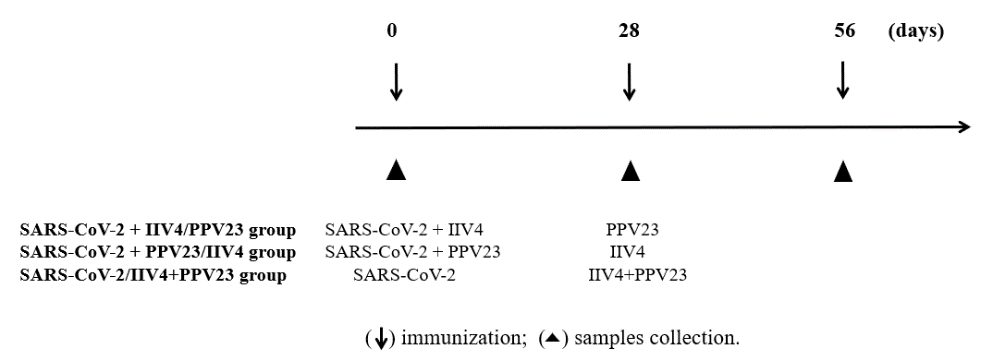


**Supplementary material 1. Schemes for immunization and sample collection.**

Blood samples were collected before and 28 days after immunization for specific antibody testing for each type of vaccine.

**a**


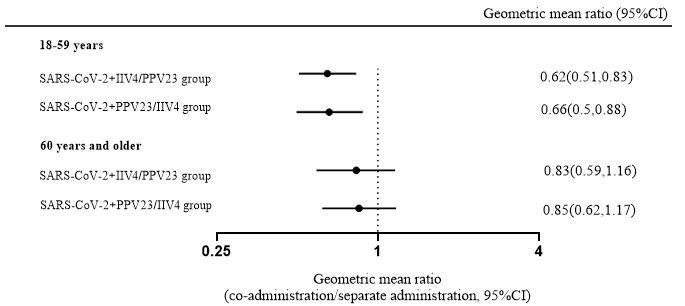


**b**


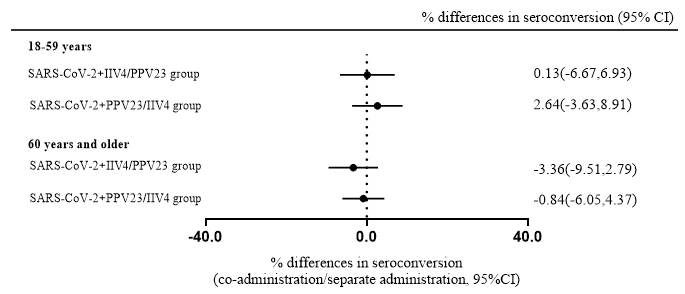


**Supplementary material 2.** **Non-inferiority for SARS-CoV-2 neutralizing antibody (a) geometric mean titer ratios and (b) seroconversion rate of co-administration versus separate administration.**

**
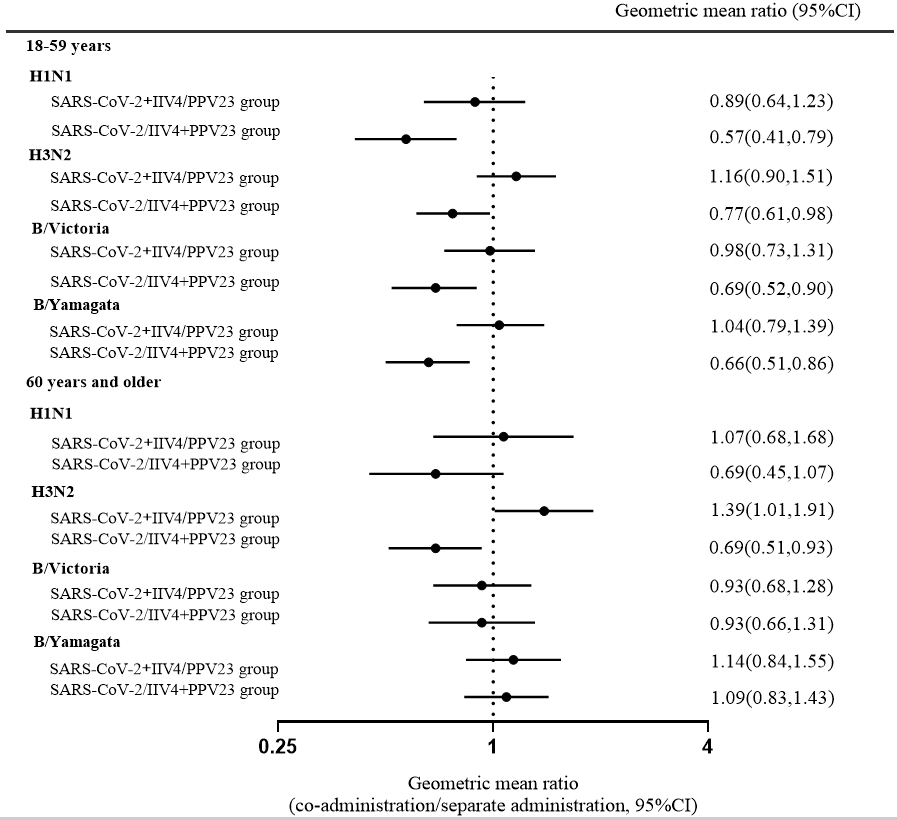
a**


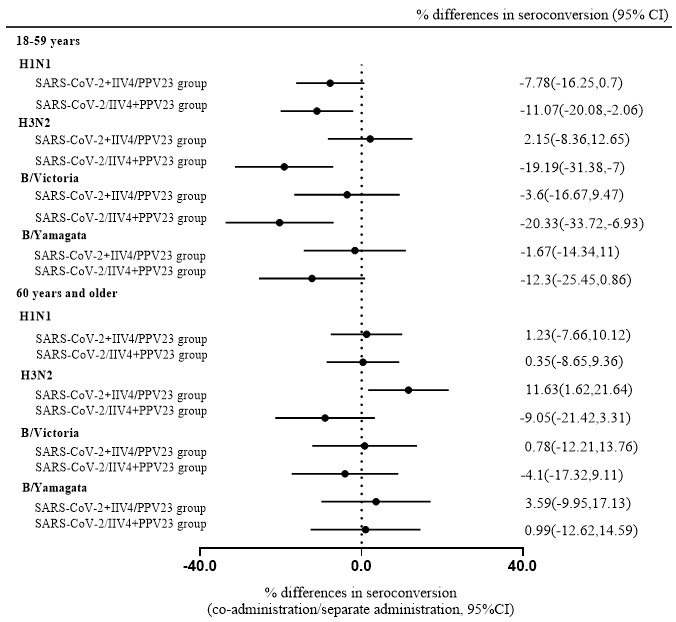


**b**

**Supplementary material 3. Non-inferiority for influenza** **haemagglutination inhibition (a) geometric mean titer ratios and (b) seroconversion rate of co-administration versus separate administration.**

**
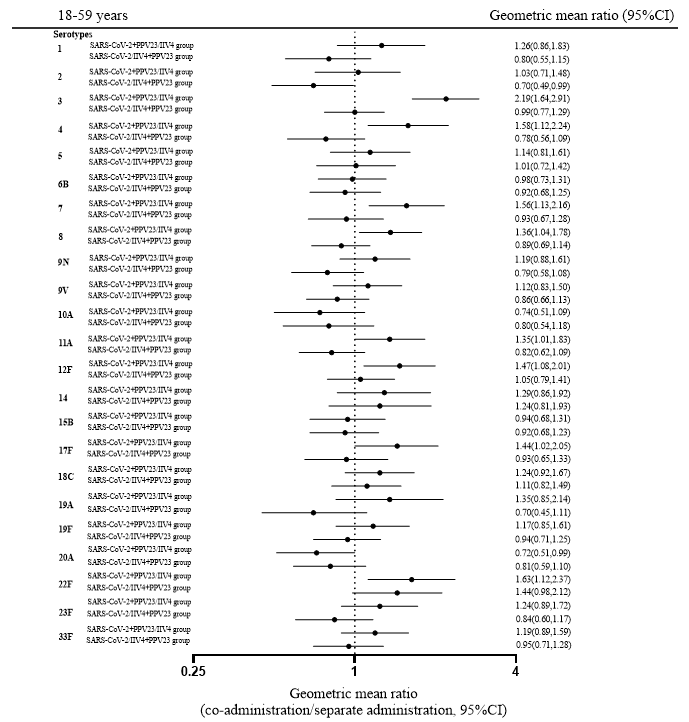
a**

**
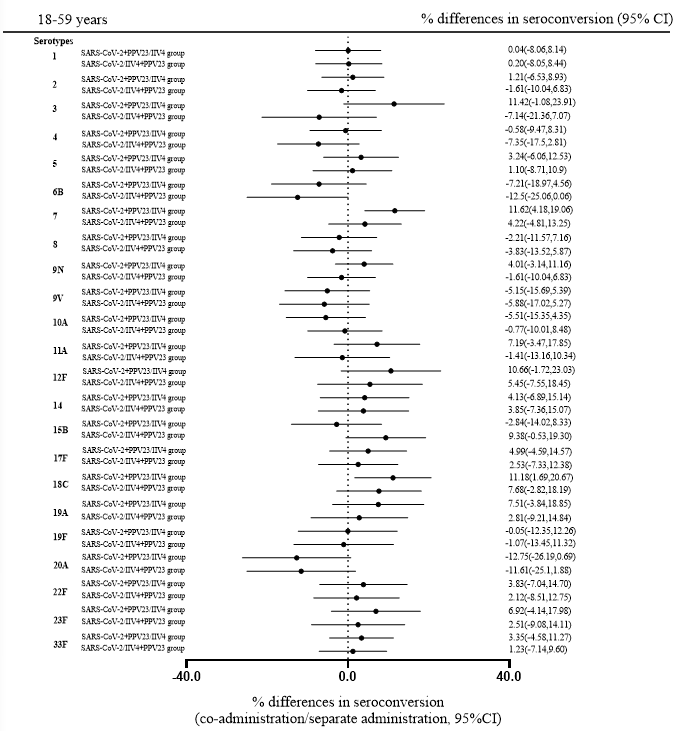
b**


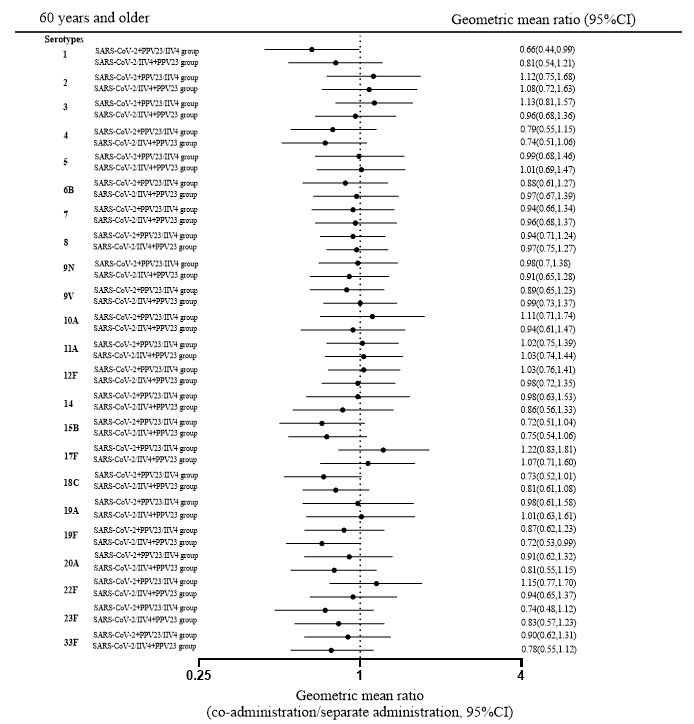
**c**


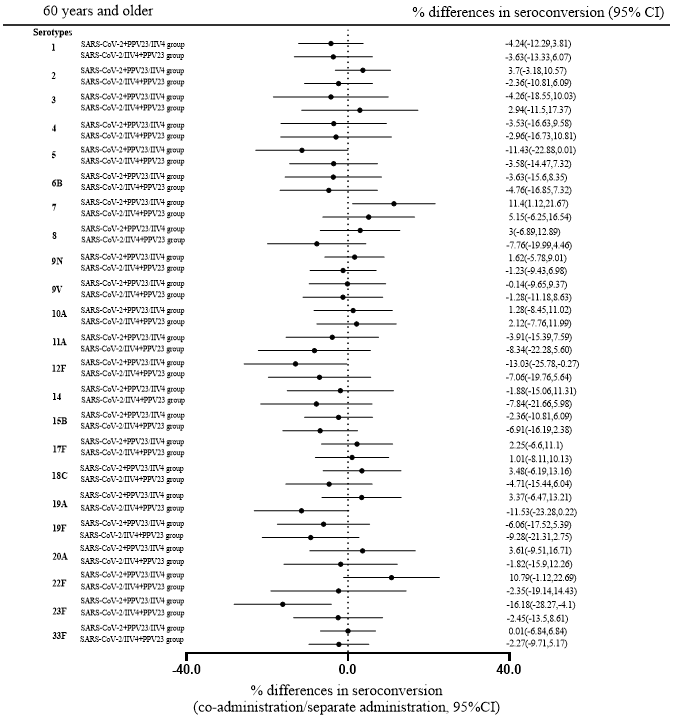
**d**

**Supplementary material 4.** **Non-inferiority for 23-valent pneumococcal polysaccharide vaccine (a and c) IgG geometric mean concentrations (GMC) ratios and (b and d) seroconversion rate of co-administration versus separate administration.**
